# Left-Sided Colonic Tuberculosis Presenting as Colonic Stricture: A Rare Presentation of a Common Disease

**DOI:** 10.14309/crj.0000000000000928

**Published:** 2022-11-24

**Authors:** Shabana Abdul Jabbar, B. Selvakumar, Vaibhav Kumar Varshney, Indu Sharma, Sudeep Khera, Sabir Hussain

**Affiliations:** 1 Department of Surgical Gastroenterology, All India Institute of Medical Sciences, Jodhpur, Rajasthan, India; 2 Department of Pathology, All India Institute of Medical Sciences, Jodhpur, Rajasthan, India; 3 Department of Medical Gastroenterology, Dr. S. N. Medical College, Jodhpur, Rajasthan, India

## Abstract

Gastrointestinal tuberculosis (TB) mainly presents as an ileocecal disease, and colonic TB is more often seen with terminal ileal involvement. Isolated involvement of the descending colon by TB is uncommon and usually presents with chronic colitis. An acute presentation as intestinal obstruction because of tubercular stricture of the descending colon has not been reported. We encountered a young woman who presented with features of acute bowel obstruction. On evaluation, she was diagnosed with a case of descending colon stricture with a provisional diagnosis of malignant colonic stricture. Left hemicolectomy was performed, and histopathology revealed it to be tubercular stricture. Antitubercular therapy was given for 9 months, and she is doing well at follow-up. A differential diagnosis of TB at an unusual location should always be considered even when presented with atypical symptoms, especially for patients from the endemic zone of TB.

## INTRODUCTION

Tuberculosis (TB) is one of the most common communicable diseases worldwide. It is primarily a pulmonary disease with an overall mortality of around 15%. Although extrapulmonary TB accounts for 15%–20% of all cases, abdominal TB comprises only 10%.^1^ Because of the insidious course of the disease and its nonspecific manifestations, it is difficult to establish the correct diagnosis of abdominal TB. A high suspicion should be maintained to reach a correct diagnosis in the background of risk factors for TB such as poor socioeconomic status, undernutrition, or immunosuppression, especially in endemic regions.

Colonic TB is rare, and descending colon is one of the least commonly affected sites in colonic TB.^1,2^ Only a few reports have described TB of the left colon, with most patients presenting with a chronic history of malnutrition and abdominal pain. However, left colonic TB causing a stricture and presenting primarily with bowel obstruction is rare; this is usually the presentation of colonic malignancy. Here, we present a case of stricture forming descending colon TB in a young woman who presented with large bowel obstruction.

## CASE REPORT

A 33-year-old woman presented with 2-month history of intermittent colicky central abdominal pain and altered bowel habits in the form of constipation associated with nausea, postprandial bloating, and easy fatiguability. There was worsening abdominal pain, vomiting, abdominal distension, and obstipation for the past 2 days. There was no history of anorexia, weight loss, fever, or lower gastrointestinal bleed. Her father was diagnosed 10 years earlier with abdominal TB and treated with antitubercular therapy (ATT).

Although her general clinical examination was unremarkable, the abdomen was distended with palpable bowel loops and exaggerated bowel sounds, suggestive of acute intestinal obstruction. Her digital rectal examination was unremarkable. Her laboratory parameters showed no derangement, and serum carcinoembryonic antigen was 0.65 ng/mL (reference range: 0–2.5 ng/mL).

**Figure 1.**
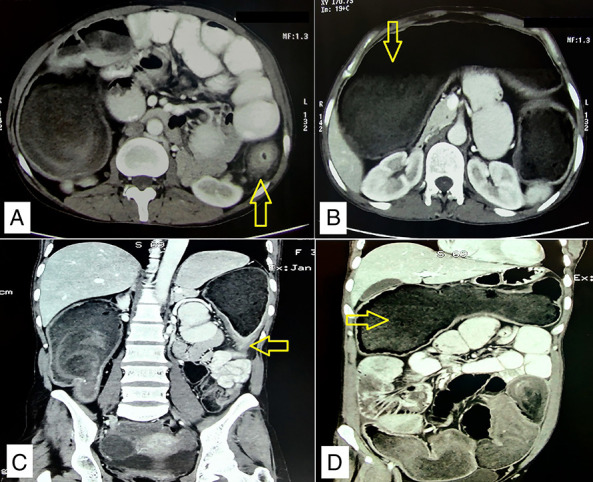
Contrast-enhanced computed tomography of the abdomen and pelvis. (A and B) Axial images showing descending colon stricture (arrows) with dilated proximal bowel. (C and D) Coronal images showing descending colon stricture with dilated transverse colon.

A contrast-enhanced computed tomography (CECT) of the abdomen showed a short-segment circumferential thickening and stricture in the proximal descending colon with adjacent fat stranding and multiple enlarged mesocolic lymph nodes. The proximal large bowel was grossly dilated (∼6 cm) with fecal-loaded transverse colon, ascending colon, and cecum (Figure 1). The diagnosis of left colonic stricture, likely because of inflammatory stricture, was kept as the first differential, followed by malignancy. After giving sequential rectal enemas, a colonoscopic examination was attempted to take a biopsy and/or to attempt balloon dilatation/stenting of the stricture, but it was unsuccessful because of inadequate clearance of the distal colon.

**Figure 2.**
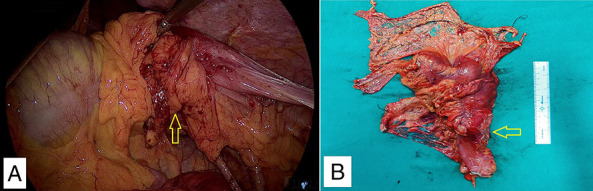
Intraoperative images: (A) laparoscopic image of descending colon stricture (arrow) with proximal transverse colon dilatation. (B) Postoperative left hemicolectomy specimen showing descending colon stricture (arrow).

As the patient was in acute large bowel obstruction, she was planned for an emergency left hemicolectomy. After a staging laparoscopy to rule out peritoneal and liver metastases, a 5 × 3-cm stricture was noted in the proximal descending colon with a dilated transverse and ascending colon. During the laparoscopic phase, the left colic artery was dissected at its origin from the inferior mesenteric artery, and its low ligation was performed. The inferior mesenteric vein was ligated at the same level. The principle lymph nodes at the base of inferior mesenteric artery were separately dissected. The descending colon was mobilized laparoscopically by a medial to lateral approach. The gastrocolic ligament was divided, and the splenic flexure was completely mobilized. In view of the grossly dilated transverse colon, the middle colic vessels could not be adequately visualized. Hence, the procedure was converted to open using a midline laparotomy incision. The transverse mesocolon was mobilized, and the left branch of the middle colic vessels was identified and ligated. The proximal colon was clamped and divided at the midtransverse colon, and distally, it was divided at the junction of descending and sigmoid colon. The proximal colon was then evacuated completely in a controlled manner using intraoperative on-table colonic lavage using ∼3-L lukewarm saline till clear returns were ensured. The colonic wall edema, as well as diameter, was significantly decreased. In view of the stable hemodynamic parameters and optimal nutritional status, a primary colonic reconstruction was deemed safe, and a handsewn, 2-layered, colocolic anastomosis was made (Figure 2).

**Figure 3.**
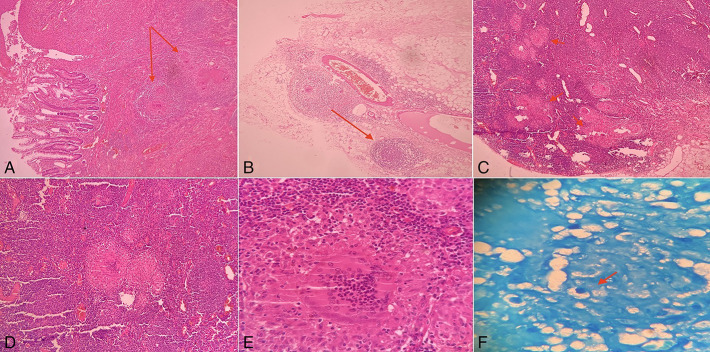
Histopathology examination: (A) section of intestine showing dense chronic inflammatory infiltrate and multiple well-formed epithelioid granulomas in the submucosa (arrows) (H&E, 4×). (B) Presence of epithelioid granulomas in pericolic fat (arrow) (H&E, 10×). (C) Multiple epithelioid granulomas in mesenteric lymph node (H&E, 4×). (D) High-power view of epithelioid granuloma (H&E, 10×). (E) High-power view of epithelioid granuloma (H&E, 40×). (F) Ziehl-Neelsen stain highlighting the presence of acid-fast bacilli (arrow) (Ziehl-Neelsen stain, 100×).

Gross examination of the resected specimen revealed a circumferential stricture in the descending colon, which was solid, gray-white, and homogenous in texture. Histopathology of the strictured area revealed ulcerated mucosa, submucosal granulomatous inflammation with numerous epithelioid cell granuloma, and Langhans giant cells surrounded by areas of necrosis. All 21 lymph nodes identified from the specimen showed epithelioid cell granulomas and Langhans type of giant cells with few foci of necrosis. Ziehl-Neelsen stain showed acid-fast bacilli (Figure 3). Hence, a diagnosis of tubercular stricture of the left colon was confirmed.

**Figure 4.**
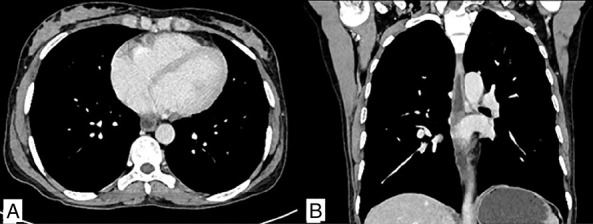
Contrast-enhanced computed tomography of the thorax. (A and B) Axial and coronal images showing no abnormal findings.

She had an uneventful postoperative recovery and was discharged on a regular oral diet on the fourth postoperative day. A CECT thorax was performed in the postoperative period to rule out evidence of coexistent pulmonary TB, which was essentially normal (Figure 4). She was started on ATT with 3 months of intensive phase comprising 4 drugs (isoniazid-H, rifampicin-R, pyrazinamide-Z, and ethambutol-E) followed by 6 months of continuation phase with HRE. After 9 months, she is doing well with no side effects of ATT and no evidence of TB elsewhere on a follow-up scan.

## DISCUSSION

India has the highest prevalence of TB worldwide, accounting for ∼26% of all cases.^3^ The risk factors are immunosuppression, undernutrition, low socioeconomic status, overcrowded living conditions, and alcohol and tobacco abuse. Although the patient in this report belonged to a middle-class family with good social conditions, she had a contact history of TB in the family.

TB is primarily a disease of the lungs, and although extrapulmonary TB can affect 15% of cases, luminal gastrointestinal TB is encountered in nearly half of the population among them.^1,4^ The terminal ileum is the most commonly involved anatomical location in gastrointestinal TB, and the cecum is the most commonly involved site in colonic TB.^3^ TB involves the abdomen through various routes, including ingestion of infected food or milk (primary intestinal TB) or infected sputum (secondary intestinal TB), hematogenous spread from distant tubercular focus, contiguous spread from infected adjacent foci, or through lymphatic channels. The relatively higher lymphatic tissue and longer transit time because of the ileocecal valve could explain the higher incidence of TB in the terminal ileum.

The isolated involvement of the colon by TB is rare (∼10%), with the most common sites being the cecum, ascending, or transverse colon.^3^ The descending colon is the least common site to be involved because of TB, and over the past 2 decades, isolated or segmental TB affecting it exclusively has been reported very sparsely (Table 1).^2,5–14^

**Table 1.** Various cases of descending colon TB with varied presentation

S. No.	Study	Cases of descending colon TB	Age (yr)/sex	Clinical presentation	Colonoscopy with biopsy	CECT abdomen	Management
1	Nagi et al^2^	2	NA	NA	Yes	NA	ATT/surgery
2	Medina et al^5^	1	71/F	Rectal bleeding and tenesmus	Yes	NA	ATT
3	Shah et al^6^	2	NA	Abdominal pain, anorexia, and weight loss	Yes	NA	ATT
4	de Jesus et al^7^	1	10/M	Abdominal pain, diarrhea, and weight loss	Yes	Splenic flexure stenosis with intestinal obstruction	ATT followed by surgical resection
5	Alvares et al^8^	4	NA	Abdominal pain, anorexia, weight loss, fever, and diarrhea	Yes	NA	ATT
6	Yu et al^9^	1	80/M	Nil (routine medical examination)	Yes	No particular findings	ATT
7	Hajong et al^10^	1	46/F	Abdominal distension, rectal bleeding, fatigue, and constipation	Yes	Descending colon stricture	ATT followed by surgical resection
8	Wang and Yu^11^	2	34/F, 28/M	Altered bowel habits, rectal bleeding, and tenesmus	Yes	NA	ATT/surgery
9	Philpott et al^12^	1	82/F	Abdominal pain, weight loss, and diarrhea	No	Sigmoid diverticulitis	Surgical resection
10	Chakinala et al^13^	1	36/F	Abdominal pain, loose stools, and weight loss	Yes, negative	Descending colon stricture	ATT followed by surgical resection
11	Gompertz et al^14^	1	55/M	Fatigue, anorexia, and weight loss	Yes	Rectal and omental thickening, ascites, and abdominopelvic adenopathies	ATT
12	Present case	1	33/F	Abdominal pain, obstipation, abdominal distension, and vomiting	No	Descending colon stricture with proximal bowel dilatation	Surgical resection followed by ATT

ATT, antitubercular therapy; CECT, contrast-enhanced computed tomography; F, female; M, male; NA, not available; TB, tuberculosis.

The presentation of isolated colonic TB is often nonspecific such as a long-standing history of abdominal pain, anorexia, weight loss, and altered bowel habits. Around 10%–15% of patients may have rectal bleeding and awareness of abdominal lump.^5,15^ Hence, it is usually misdiagnosed as chronic inflammatory bowel disease or malignancy. The presentation of isolated colonic TB as an acute intestinal obstruction is uncommon and usually involves the right colon.^16,17^ On the contrary, left-sided colonic TB presenting as acute large bowel obstruction because of tubercular stricture, as seen in our patient, has been sporadically reported.

Wang and Yu described acute distal bowel obstruction because of descending colon mass in a young man. He underwent urgent laparotomy and diversion colostomy. He was diagnosed with abdominal TB on nodal biopsy and was further treated with ATT, followed by colonic resection and restoration of bowel continuity performed in second-stage surgery.^11^ de Jesus et al reported stenosing left-sided TB colitis in a 10-year-old boy with chronic abdominal pain and malnutrition. Initially, he was managed medically; however, he required surgical resection for a perforated lesion.^7^ Hajong et al reported a case of descending colon TB in a 46-year-old woman with rectal bleeding and chronic constipation. She was initially managed with ATT; however, a colonic resection was performed later because of persistent bleeding per rectum and altered bowel habits.^10^

A diagnosis of colonic TB is suspected on radiology by the presence of ileocecal stricture with other features such as necrotic lymphadenopathy and pulled-up cecum, in the background of a clinical history, suggestive of colitis with long-standing abdominal cramps, altered bowel habits, and loss of weight. Although the sensitivity is low, a preoperative diagnosis of TB is confirmed on colonic biopsy showing submucosal and mucosal caseating granulomas containing Langhans giant cells with acid-fast bacilli on Ziehl-Neelsen stain.^3^ In the present case, the preoperative CECT abdomen revealed stricture of descending colon akin to malignancy with acute obstruction, no other evidence of abdominal TB, and the preoperative chest radiograph revealed no pulmonary lesions. A colonoscopy-guided biopsy was not feasible because of symptoms of obstipation; furthermore, an attempt to perform balloon dilatation/stenting of the stricture was also unsuccessful because of inadequate clearance of the distal colon even after giving rectal enemas. Hence, she was provisionally diagnosed with a malignant obstructing stricture of descending colon and underwent a left hemicolectomy.

In the presence of risk factors such as hailing from endemic regions such as India, self-history of pulmonary TB, or contact with a patient of TB, a high degree of suspicion and possibility of TB should be kept in the differentials. However, this would have been beneficial in the early course of the disease because she could have been evaluated colonoscopically and administered ATT preoperatively. This may have averted the above complication and avoided a surgical resection.

The mainstay of treatment for colonic TB is ATT as per the National TB Elimination Programme guidelines. A fixed-dose combination of isoniazid, pyrazinamide, rifampicin, and ethambutol is administered with a dosage decided based on the patient's weight. The treatment is to be administered for at least 6–12 months.^3,4^ Surgical treatment is reserved for refractory cases of TB who continue to have chronic malnutrition because of malabsorption or chronic abdominal pain with altered bowel habits affecting the quality of life. In general, the management of left-sided mechanical obstruction of the colon varies from primary resection with or without a primary reconstruction with or without a stoma; or a primary colonic diversion using an ileostomy or a colostomy; or endoluminal colonic stenting as a bridge to elective surgery. The patient's preoperative parameters such as serum albumin, body mass index, and intraoperative parameters such as blood loss, duration of surgery, hemodynamic status, inotropic support as well as the status of bowel edema are the factors that should be taken into consideration before deciding on the optimal management strategy. Surgery is also warranted in patients with acute complications such as intestinal perforation and obstruction. However, postoperative ATT should be administered as per standard protocol or resumed if already received preoperatively.

Regular follow-up visits are advised to ensure drug compliance and completion of the course. Most patients respond clinically to ATT and have complete resolution of symptoms and lesions. Hence, routine imaging or colonoscopy is not recommended in follow-up unless clinically warranted, as in cases of recurrent symptoms of abdominal pain or obstruction.^15^

In conclusion, isolated stricture of the descending colon is a rare presentation of abdominal TB, often misdiagnosed as a malignancy or inflammatory bowel disease. A history of chronic malnutrition and altered bowel habits in the background of a strong contact history of TB should prompt the physician to maintain a high index of suspicion and include colonic TB in the differentials. Surgery should be as conservative as clinically feasible; however, in the background of suspicion of malignancy, a radical surgical resection may be justified to ensure an R0 resection with adequate lymph node clearance.

## DISCLOSURES

Author contributions: SA Jabbar, B. Selvakumar, VK Varshney, I. Sharma, S. Khera, and S. Hussain were involved in diagnosing and managing the patient. SA Jabbar, B. Selvakumar, and VK Varshney provided the important intellectual content to the manuscript. All authors have read the final version of the manuscript and approved it. VK Varshney is the article guarantor.

Financial disclosure: None to report.

Informed consent was obtained for this case report.
